# Recommendations for mobile apps for mental health treatment: Qualitative interviews with psychiatrists

**DOI:** 10.1177/20552076251325951

**Published:** 2025-03-17

**Authors:** Harleen Gill, Catriona Hippman, Saskia Hanft-Robert, Lena Nugent, Ondřej Nováček, Mostafa M. Kamel, Deirdre Ryan, Regina Demlová, Michael Krausz, Katarina Tabi

**Affiliations:** 1 Department of Psychiatry, 8166 University of British Columbia, Vancouver, Canada; 2 8163 BC Reproductive Mental Health Program, BC Women's Hospital, Vancouver, Canada; 3 Department of Department of Obstetrics & Gynaecology, 8166 University of British Columbia, Vancouver, Canada; 4 Department of Medical Psychology, 37734 University Medical Center Hamburg-Eppendorf, Hamburg, Germany; 5 Centre for Psychosocial Medicine, 37734 University Medical Center Hamburg-Eppendorf, Hamburg, Germany; 6 Department of English and American Studies, 37748 Masaryk University, Brno, Czechia; 7 Department of Psychiatry, 68781 Tanta University, Tanta, Egypt; 8 Department of Pharmacology, 37748 Masaryk University, Brno, Czechia; 9 BCCH Centre for Mindfulness, 37210 BC Children's Hospital, Vancouver, Canada

**Keywords:** Mental health, mobile app, mHealth, qualitative, psychiatrists, app design, app content, patients

## Abstract

**Background:**

The number of mobile apps tailored for people living with mental health conditions has increased tremendously. However, the majority of the existing apps are not evidence-based and are being developed by teams without mental health expertise.

**Objective:**

We aimed to explore psychiatrists’ perceptions of what they and their patients need in a mental health app and eventually inform the design of future mobile apps in this area.

**Methods:**

Semi-structured interviews were conducted with psychiatrists (*N *= 18) from three European countries: Austria, the Czech Republic, and Slovakia. Content analysis using inductive and deductive coding was used to analyze the interviews.

**Results:**

Four major themes were deductively identified: current system, gaps in the current system, recommendations for a mobile app, and promoting app use. Psychiatrists provided a comprehensive list of app features they suggested would be helpful. Of particular importance seemed to be enabling patients to self-monitor various aspects of their lives and including an emergency plan. Participants also emphasized that the app should be positive and motivating for patients to use, with some suggesting that users be able to communicate with other users for support. Within the theme of “current system,” a common topic was the current shortage of psychiatrists and the feelings of time pressure amongst existing psychiatrists.

**Conclusions:**

The results of this study can be used by software developers to inform future designs of mental health mobile apps, which will hopefully translate to a greater availability of evidence-based apps that address clinical needs.

Mental health is a critical component of overall health, and its neglect can lead to severe negative outcomes. However, accessing services to address mental health challenges can be quite difficult. For instance, the World Health Organization^1^ declared a worldwide psychiatrist shortage, reporting that many countries had less than three psychiatrists per 100,000 people. In order to bridge the gap between the mental health services available and the need for services, e-mental health resources, including mobile apps, are increasingly being used.

In addition to increasing accessibility to care, mental health apps can enhance the quality of existing care when used in conjunction with visits to mental health professionals.^2^ The European Federation of Psychologists’ Associations’ eHealth taskforce reported that such blended use could help people change their behaviors by tracking them in an app and being prompted to practice certain strategies.^3^ In addition to the benefits of skills practice and psychoeducation, individuals have been found to self-disclose more digitally than verbally.^4,5^ Thus, psychiatrists may receive more accurate and higher-quality information by examining patients’ app data than solely relying on self-reports during appointments, allowing psychiatrists to better understand their patients’ lived experiences and optimize their treatments.

The need for e-mental health resources became even more apparent during the COVID-19 pandemic.^6,7^ Santomauro et al.^8^ showed that the pandemic challenged people's mental health globally as their review of 204 countries and territories found an increased prevalence of anxiety disorders and major depressive disorders. Given this and fears around the COVID-19 pandemic, it is not surprising that people turned to e-mental health resources for help. In fact, 238 million installs of mobile wellness apps occurred in April 2020 worldwide, a steep increase from the 87 million and 78 million installs that occurred in January 2020 and February 2020, respectively.^9^

However, finding and identifying a high-quality, evidence-based app is not easy. The overall number of available mental health apps has been rapidly increasing,^10^ but most apps are not being developed in collaboration with healthcare professionals, nor are they rooted in scientific evidence.^3,11–13^ Tabi et al.^12^ showed that less than 15% of health apps were developed with the involvement of a health expert, and the literature suggests that out of thousands of mental health apps, less than 0.1% were clinically validated.^14,15^ This is concerning as patients could be adversely affected by using an app that is not grounded in evidence as it may provide misinformation.^14,16^ The development of future mobile apps should be informed by scientific evidence and include the perspectives of both patients and healthcare professionals.

We focused on exploring psychiatrists’ perceptions of what they and their patients need in a mental health mobile app to help developers create evidence-based apps. In addition to this primary goal, we explored the state of the current healthcare system and potential motivators for the use of future apps by patients and psychiatrists.

## Methods

### Study design

The study comprised semi-structured qualitative interviews with 18 psychiatrists. The content of the interviews primarily focused on psychiatrists’ perceptions and suggestions for the future development of mobile apps tailored for their patients.

A qualitative approach was chosen which allowed an in-depth and flexible exploration of the participants’ perspectives, experiences, and suggestions for the development of mobile apps for supporting their patients’ mental health treatment. The interviews were conducted by a researcher with training and experience in qualitative research (KT).

This project received ethics approval from the Ethics Committee of the Faculty of Medicine, Masaryk University (REB approval: 92017). The reporting of methods is in accordance with the consolidated criteria for reporting qualitative research (COREQ).^17^

### Recruitment

Psychiatrists were recruited from six different locations across the Czech Republic, Austria, and Slovakia. Participants were recruited purposefully for one-on-one interviews.^17^ Purposeful sampling is a common technique in qualitative research to select information-rich participants with particular knowledge or experience in the area of interest to answer the research question in the best possible way.^18–22^ Only individuals who were at the time of the study actively practicing and fully trained psychiatrists in an inpatient or outpatient mental healthcare setting were included. All individuals who were mental healthcare providers but not fully trained psychiatrists were excluded from study participation. Snowball sampling was used to find and recruit eligible study participants.^18,23^ The snowball sampling began with KT approaching a colleague via email, and then the method of approach by individuals was a combination of email, face-to-face, and telephone. A maximum variation sample^18^ was recruited with a focus on diversity of ages, years of working experience, and genders. Data collected from the interviews were reviewed to ensure anonymity.

KT introduced herself to the participants before the interview started, including information about her academic institution, lab, credentials, basic information about the study, and why it was being conducted. Participants were informed about the study's procedure and details prior to the interview in both oral and written form in their native language (Czech Republic, Slovakia) or in English (Austria). They also received an informed consent form in their native language or in English.

### Researchers’ characteristics

Data collection and analysis were led by HG (female), KT (female), SHR (female), and MK (male). HG has a B.Sc. in behavioral neuroscience and experience with developmental and clinical psychological research, including conducting semi-structured interviews and coding behavioral observations. KT is a research fellow, with expertise in e-mental health, psychiatry, mindfulness, and neuropsychopharmacology, and has experience in content design and analysis of mental health software for research purposes. SHR is a M.Sc. psychologist and PhD student with many years of experience in developing and conducting semi-structured interviews and qualitative data analysis. MK is a professor of psychiatry with comprehensive expertise in e-mental health research as well as qualitative research and data analysis.

### The qualitative interview

A semi-structured interview guide^24^ was developed by KT and a graduate student in consultation with MK and other team members. It was discussed and refined several times by an interdisciplinary research team, and the final guide was pilot tested in two interviews with psychiatrists. Feedback from the two psychiatrists informed the refining process, with minor adjustments subsequently made to the guide. The pilot interviews were not part of the study's sample.

All interviews were conducted one-on-one in person at the psychiatrists’ offices by KT according to the interview guide. The participant and interviewer agreed upon the time and place of the interviews during their initial contact. The interviews were held in English, Slovak, or Czech, based on the preference of the participant. Each interview lasted between 30 and 60 minutes, and no repeat interviews were conducted. Participation in the study was voluntary and not remunerated. The interviews were audiotaped using two sound recorders. KT wrote basic field notes during and after the interview; however, they were not used during the analysis as the team agreed that the recordings provided sufficient information.

At the beginning of the interview, participants were encouraged to share their personal experiences and perspectives, rather than scenarios suggested in clinical guidelines. The interview guide consisted of two main parts. The first part asked how the participants perceived the current system. Questions included the following two topics: the routine procedure in their clinical practice when introducing a patient to a new psychiatric medication and gaps and challenges within the current system of care. The second part asked how to overcome the perceived gaps and challenges in mental health services. Psychiatrists were encouraged to share their ideas and suggestions for the development of a future mobile app that could support patients during the treatment process. They were asked what features and characteristics they would suggest including in such a mobile app. The interviewees were encouraged to come up with their own ideas. In cases where participants had no more suggestions, the interviewer mentioned some existing features of apps and inquired about their opinion of those features. At the end of the interview, participants were asked how patients and physicians could be motivated to use an e-mental health app as something new in their lives.

### Data transcription and analysis

Audio recordings were transcribed verbatim into text format using the software Sonix and Otter. To ensure that all interviews could be coded using a single language, interviews that were originally conducted in Czech or Slovak were translated into English by KT, ON, and MMK, who are fluent in all three languages. Finally, all transcripts were checked manually for accuracy and corrected when necessary by research assistants.

The analysis of the interview data was done by HG and SHR in close consultation with KT using the software MAXQDA 2018.^25^ The researchers conducting the data analysis were blind to the identity of the participants. All 18 transcripts were read multiple times by the researchers before coding. Data analysis of the transcribed interviews followed the method of structuring content analysis.^26–28^ The structuring content analysis approach aims at reducing, summarizing, and structuring a large amount of qualitative data by creating a coding frame, consisting of main themes and subthemes. A combination of inductive and deductive coding was used.^27,28^ Deductive themes were derived from the interview guide and research questions and formed the main themes “current system,” “gaps in the current system,” “recommendations for a mobile app,” and “promoting app use.” Topics brought up by interviewees were added as inductive subthemes to the coding frame. To ensure intersubjective comprehensibility and credibility, HG and SHR each coded two transcribed interviews separately and discussed the themes afterward.^19^ Based on this and the deductive themes, a first coding frame was developed. Subsequently, the remaining interviews were analyzed by HG and SHR in close collaboration. The existing coding frame was supplemented and revised with additional inductive subthemes by HG and SHR in close consultation with KT. The final coding frame was presented to, and discussed within, a large interdisciplinary research team to ensure intersubjective reproducibility and comprehensibility.^19^ Study participants did not provide feedback on the findings.

## Results

The study recruited 18 psychiatrists (females = 11, males = 7) from six different locations across the Czech Republic (*n *= 9), Austria (*n *= 6), and Slovakia (*n *= 3). Participants all identified as White. All but one participant disclosed their age (*mean age *= 41; *range *= 29–60). No participants dropped out or refused to participate.

Four main themes, 13 subthemes, and 14 secondary subthemes were identified. The main themes were created deductively based on the research questions and include *the current system of care and resources*, *problems with the current system*, *recommendations for a mobile app*, and *promoting app use.*

### Primary findings: recommendations for a mobile app

Participants’ recommendations were separated into two main subthemes: design and features. An overview of all design and feature recommendations is presented in Table 1. More detailed explanations of the recommendations made by participants, including their quotes, are included in Table S1.

**Table 1.** List of recommendations for app features and design.

Recommendation for App—Topic	Recommendation for App—Subtopics	More Details
Features and Content		
Information about medication and other treatment options	Type, how medication works	
	Effects	Expected positive effects Creating realistic expectations and correcting false beliefs How long it will take to experience the effects
	Side effects	Only most common Coping tips
	Risks of using non-prescribed substances while on medication	
	Treatment interruptions	
	Information about alternative treatment options	
Information about mental illness		
Additional sources of information	FAQ	
	Links to external websites/resources	
Tracking	How to track	Diary entries Scales for rating Automatic data collection Flexibility in tracking
	Symptoms, health outcomes, and health behaviors	Anxiety symptoms Appetite and food intake Libido Physical activity (and correlation with mood) Physical condition (e.g. rate their pain) Mood Sleep Socializing behavior Stress Weight Moments to pause/be alone/spiritual engagement
	Person's environment	Correlation between light level and mood
	Non-prescribed substances	Alcohol consumption Cigarette use
Reminders	Doctor's appointments	
	Reminder to take medication	
	Reminder to eat	
	Reminder to drink water	
Appointment booking system		
Sharing data and reports	Ability to share app data	Access for doctor Access/notifications for family
	Ability to analyze app data and provide feedback	
	Access to medical records	
Communication options	Forums/chat rooms with other patients/doctor/family/social workers	Possibly moderated/supervised
	One-on-one messages with doctor	Notify to contact doctor
	Connection to call center	
Plan out their day		
Life path and goals		
Emergency plan	Crisis button	
	Individual warning signs	
	Include family members	
	Cooperation with a professional	
	App contacts emergency contact (given previous consent)	
Design		
Simple, easy, not overwhelming		
Positive and motivating	Success stories of other patients	
Pictures		
Video explanations		
Interactive		
Story or game		

### Exploratory analyses

#### The current system of care and resources

##### Explain treatment process

Many psychiatrists reported that as part of their practice, they aim to explain the patient's condition to them, as well as the suggested treatment process and basic information related to the new medication: “Generally, I start with the diagnosis. I describe the diagnosis, I explain to the patient which medication they are going to use and why, and how it works. I try to comfort the patient by saying that we are not starting with medication that has a strong effect. When prescribing, for example, antidepressants, I tell the patient that the effect is not imminent. I tell them when we expect the effect to occur. […] And if they have any questions, I answer them” (IP_Q).

Some of the interviewees highlighted that in situations where it is relevant, they find it important to inform the patient that medication may only be one step of the treatment process and that other treatment strategies will be added to support the patient's long-term well-being: “We tell the patients that medication will help them sleep, increase their appetite, etc., but that it is just the beginning of the path to recovery, or part of it. Medication prepares the foundation, but the main thing is the psychotherapy and the work with the patient” (IP_I).

Psychiatrists reported they aim to explain, as a minimum, the most important things about the medication treatment to the patient. Information that was mentioned involved effects, including both what the medication can help with and what it cannot do, common side effects, how the medication works, and the waiting period until it starts to work*.*

##### Address patient's expectations and false beliefs

As part of discussing the new treatment, the participants stated that they asked their patients about their expectations and concerns, demonstrating that they were highly aware of unrealistic expectations or false beliefs that patients may have (e.g. becoming addicted to a non-addictive medication). They reported addressing these: “I always ask my patient about their expectations. The patient tells me what they think, and I tell them what is real and what is not,” (IP_P), and “I tell them that the medication can improve their mental state, but that no medication can solve their problems. […] For example, the antidepressants give the person more energy so they are able to change their life, but the medication won’t make the change itself. When I see people who have bad relations with their family, wife, I say that the medication won’t solve that” (IP_I).

##### Contact psychiatrists for additional needs

Interviewees said they encourage patients to contact them if any problems or questions arise. However, some of them mentioned receiving phone calls or social media messages from patients whether they had explicitly encouraged this sort of contact or not. Emails and messaging over social media appeared to be unusual; phone calls were the contact method of choice for patients: “I give them my number so that the patient can call me in case the side effects appear, or if they encounter something that they were not informed about, or if they have some other questions” (IP_N).

### Problems with the current system

#### Demand for psychiatrists exceeds supply

The most frequently cited problem by psychiatrists was that the patient demand was greater than the supply of psychiatrists. They emphasized that wait times for patients were longer than they should be, highlighting that the shortage of psychiatrists and the lack of time that existing psychiatrists had went hand-in-hand: “In the outpatient care there are not enough psychiatrists. And when a patient makes an appointment, they have to wait two or three months” (IP_M).

Participants described how lengthy appointments with each patient are not possible, especially in outpatient care, which sometimes leaves patients with unanswered questions that can be addressed by contacting the doctor in between appointments or at their next appointment: “No, the patient does not get as much time as he would want. In my care, we have our meetings last 30 minutes and patients would like to have more but this is not possible. Some of them call me 3 times a day: ‘This has happened to me, what should I do?’, or ‘I got a rash, could it be a side effect of the medication?’” (IP_P).

#### Problematic package insert

One of the first sources of written information for patients about new medication is the package inserts that come inside medication packages. However, most participants’ comments about these information sources were negative, with some of them mentioning that they discouraged their patients from reading these package inserts altogether. A common reason for this was that the inserts were not appropriate for patients. Rather than focusing on the information most helpful for the patient, the inserts focus on including information that protects the pharmaceutical company making the medication. For example, often the inserts have an extensive list of all potential side effects, including extremely rare ones, without appropriately explaining the likelihood of these side effects. This, according to our participants, can be daunting for some patients and lead to challenges with trusting medication. Other reasons that doctors in our study cited for disliking the package inserts included them not being user-friendly: “The package inserts are usually difficult to understand and don’t really offer much information” (IP_L).

#### Difficulty identifying credible information

Many study participants also discussed problems with information available online. The common theme was that there are a massive number of sources online; however, the majority of them do not contain credible and accurate information: “Because nowadays people browse the Internet and read various information which is not verified. And they have a problem making distinctions between verified and unverified information. If I could tell them where they could read the verified information, it would help me” (IP_O).

### Promoting app use

In the following, a distinction is made between how the app can be promoted amongst patients, practitioners, and both.

#### Promoting amongst patients

##### Have the psychiatrist recommend it to the patient

“If the doctor was motivated to use it, they would then motivate the patient. I think that if the doctor recommends the app to the patient, it is the best advertisement” (IP_K).

##### Present as a guide through their treatment

“I would present it to them as a complex guide through their treatment. […] Inform them that, for example, the antidepressants might in the beginning have more side effects which will then decrease. Maybe some risks of using the medication and drinking alcohol. And that they can monitor their treatment progress, what effect the medication had on them. When they have their next appointment with the doctor, it would help if they had this kind of information accessible” (IP_J).

#### Promotion among practitioners

##### Time saving

Some participants stated it should be emphasized that the app will make practitioners’ practice more efficient: “The first thing I thought about was the time difficulties […] and if the professional knew it would save their time and help the patient, then they would agree to it” (IP_O).

##### Rewards for practitioners

Some participants mentioned it would help to reward or compensate practitioners for using the app: “Health insurance companies should include it in their paid services and they should tell the doctor that if they are going to use it, if they are going to be in contact with patients through the internet, then they are going to be paid for it” (IP_I).

#### Promoting among both practitioners and patients

##### Supported by a well-known institution

Interviewees suggested that in order to increase confidence in the app and motivate individuals to use it, the app should be affiliated with or supported by a respected institution: “Also, it might be good if the app was under the auspices of some authority or some health institution, or insurance company. […] Maybe if it was supported by some university. So, the people know where it came from” (IP_K).

##### Evidence-based effectiveness

In terms of increasing trust in the app and, accordingly, its usefulness, study participants mentioned providing scientific evidence of the app's effectiveness: “Maybe to tell them that there are some studies that show that using the app leads to better results in treatment, less hospitalization, etc.” (IP_M).

## Discussion

This study explored European psychiatrists’ perspectives and suggestions regarding the beneficial features of mental health mobile apps for their patients. Of particular importance seemed to be the ability of patients to self-monitor various aspects of their lives (e.g. mood, appetite) and the inclusion of an emergency plan. Study participants emphasized self-monitoring as a critical function and listed a comprehensive list of ideas to choose from, which might be of interest to some of their patients. Some of the self-monitoring aspects from the list echoed those mentioned in previous studies: mood,^4,29^ symptoms,^30^ weight,^4^ and physical activity.^4^ The self-monitoring feature would allow patients to visually view their progress which may lead to increased adherence to their treatment plan and foster hope. Additionally, it may be beneficial for healthcare providers to see long-term data, such as a patient's symptoms over the last 6 months, so they can adjust and individualize treatment, thereby improving overall quality of care. Furthermore, previous research found that self-monitoring can increase emotional self-awareness which in turn has been linked to the reduction of symptoms and improved coping ability.^29,31^ Given the heterogeneity among patients seeking mental health treatment, it is important to note that self-monitoring may increase anxiety and lead to excessive self-monitoring for some patients with specific diagnoses. This is in line with Chan and Honey's^32^ finding that some individuals reported an increased preoccupation with negative thoughts and emotions due to self-monitoring their symptoms. To mitigate this risk, the ability to personalize the app would be essential. Specifically, the psychiatrists in our study voiced that app users should be able to choose if and what to monitor.

Another feature which stood out during the interviews was the inclusion of an emergency plan. In this proposed feature, patients would create an emergency plan that would remind them what steps to take or whom to call in a moment of crisis (e.g. when they feel suicidal or the pressure to self-harm).^33^ Participants stated that it is customary to make one so including it in the app would make it more readily available. Furthermore, some psychiatrists recommended the plan be linked to certain monitoring features, allowing the app to alert patients if there was a negative trend in their sleep or mood, for example. This suggestion echoes the finding of Lui et al.^30^ who reported that some apps would recommend therapeutic skills (e.g. muscle relaxation) or connect users to a crisis hotline depending on their self-reports. This feature could help patients to feel some agency when facing mental health challenges.

An extensive list of app features and design elements was generated by participants during the qualitative interviews. Some participants suggested finding ways to automatize data collection for the factors psychiatrists wanted to monitor to decrease the workload for patients and make the app easier and more appealing to use. Other potential benefits of this include the collection of more objective data, which could provide valuable insight into the patient's well-being and inform collaborative adjustments to enhance treatment. However, this does not come without potential barriers as this data collection would likely require the use of sensors or other external wearable technology such as that used in some ecological momentary interventions,^34^ which may deter patients. Some existing apps do automatically collect user data without the use of external technology, such as using GPS locations for context-sensing;^30^ however, such data collection also raises concerns about privacy, an issue discussed in previous mental health mobile app research.^30,32,35,36^

Another design element participants in our study continually emphasized was ensuring the app was positive and motivating for patients as they would likely be unwilling to use it otherwise. In line with creating a motivating space that fosters a sense of connection, some psychiatrists suggested allowing app users to communicate with other app users, which is consistent with findings from previous research. In one study, adolescents in Dublin placed importance on social interactions within mental health apps, suggesting that anonymous forums or being matched with another user to chat would allow them to relate to one another.^35^ Furthermore, one of the subthemes that emerged from Chan and Honey's^32^ review of 17 studies of user feedback on mental health apps was the desirability of including social platforms to facilitate social connection with others. Thus, both psychiatrists and app users appear to agree that this social interaction would have a positive impact on well-being.

There are thousands of mental health apps available,^10^ and they contain a variety of features,^37,38^ including some that our participants mentioned. One study reported the top seven features that were used in apps addressing 12 mental disorder categories: psychoeducational material, progress tracking, personalizing the app, assessment, prompts (e.g. reminders and motivational quotes), immediate in-the-moment support, and communication with a healthcare provider.^37^ While these overlap with features our participants frequently mentioned, there are some support and design features our participants mentioned that are not encompassed by the features described in this study. These include having an appointment booking system, space to plan out their day, and automatizing data collection. A different study of 27 popular apps for depression or anxiety found that while there was heterogeneity in features, common content included psychoeducation, relaxation, and assessment-related features.^38^ Our results differ from this finding in that psychiatrists did not emphasize having relaxation activities in the app. Thus, our findings overlap with features found in current mental health apps and also suggest new helpful features.

The main complaint psychiatrists in our study had regarding the current state of the mental health system was that there is a shortage of psychiatrists. According to the literature, this is not a problem unique to the countries included in our study.^1^ Evidence-based mental health mobile apps can play a vital role in addressing this gap by supporting individuals with their challenges while they wait to meet with a psychiatrist, as well as in between appointments with a mental health professional.

Lastly, psychiatrists delved into how to promote the use of the app they had described throughout their interview. A prominent subtheme which emerged was for the app to be supported by a respected institution; this would help both psychiatrists and patients trust the app as they would perceive it to be evidence-based and effective as a prerequisite for endorsement. Support for this line of thinking comes from Dragovic et al.'s^39^ finding that one of the top three requests from mental health clinicians and app users was ensuring that the app was validated. These findings also align with the American Psychiatric Association's App Evaluation Model.

### Limitations

The results of this research should be interpreted while taking several factors into consideration. One limitation to the generalizability of these findings is that data were collected in a relatively small portion of central Europe so some perspectives may be specific to these countries. However, as the literature shows that some themes such as a shortage of time and psychiatrists are relevant to other countries, both European and not, collecting data from three European countries for this study can also be looked at as a strength. Finally, the qualitative interviews in Austria were conducted in English rather than the participants’ native language. While participants self-reported being able to understand and speak English, it is possible that misunderstandings occurred or that certain opinions were not expressed because participants were not fluent or fully comfortable expressing themselves in English.

## Conclusions

This study explored the perspectives of psychiatrists regarding the beneficial features of a mental health mobile app for people living with mental health challenges. Given the lack of evidence-based mental health apps, creating one that is informed by the input of experts and evidence is a critical next step. The ideal model of health app development includes co-creation with health experts, people accessing mental health services, and app developers. However, if this is not feasible, this study offers app developers some input from health experts, which would enhance the quality of mental health apps that are developed. Future research should compare these suggestions with those of mental health professionals and patients in other countries, ensuring the patients have varying mental health diagnoses in order to capture diverse perspectives.

## Supplemental Material

sj-docx-1-dhj-10.1177_20552076251325951 - Supplemental material for Recommendations for mobile apps for mental health treatment: Qualitative interviews with psychiatrists Supplemental material, sj-docx-1-dhj-10.1177_20552076251325951 for Recommendations for mobile apps for mental health treatment: Qualitative interviews with psychiatrists by Harleen Gill, Catriona Hippman, Saskia Hanft-Robert, Lena Nugent, Ondřej Nováček, Mostafa M. Kamel, Deirdre Ryan, Regina Demlová, Michael Krausz and Katarina Tabi in DIGITAL HEALTH

sj-docx-2-dhj-10.1177_20552076251325951 - Supplemental material for Recommendations for mobile apps for mental health treatment: Qualitative interviews with psychiatrists Supplemental material, sj-docx-2-dhj-10.1177_20552076251325951 for Recommendations for mobile apps for mental health treatment: Qualitative interviews with psychiatrists by Harleen Gill, Catriona Hippman, Saskia Hanft-Robert, Lena Nugent, Ondřej Nováček, Mostafa M. Kamel, Deirdre Ryan, Regina Demlová, Michael Krausz and Katarina Tabi in DIGITAL HEALTH

sj-docx-3-dhj-10.1177_20552076251325951 - Supplemental material for Recommendations for mobile apps for mental health treatment: Qualitative interviews with psychiatrists Supplemental material, sj-docx-3-dhj-10.1177_20552076251325951 for Recommendations for mobile apps for mental health treatment: Qualitative interviews with psychiatrists by Harleen Gill, Catriona Hippman, Saskia Hanft-Robert, Lena Nugent, Ondřej Nováček, Mostafa M. Kamel, Deirdre Ryan, Regina Demlová, Michael Krausz and Katarina Tabi in DIGITAL HEALTH
