# Supplementary material for: Recommendations for mobile apps for mental health treatment: Qualitative interviews with psychiatrists
Source: Digit Health. 2025 Mar 17;11:20552076251325951. doi: 10.1177/20552076251325951 (PMC11915247; doi:10.1177/20552076251325951)
Supplement: sj-docx-1-dhj-10.1177_20552076251325951 - Supplemental material for Recommendations for mobile apps for mental health treatment: Qualitative interviews with psychiatrists [file sj-docx-1-dhj-10.1177_20552076251325951.docx]

**Table S1.** Supporting Evidence and Quotes for the Psychiatrists’ Recommendations for App Features and Design

| **Recommendation for App** | **Explanatory Notes and Quotes** |
| --- | --- |
| **Features & Content** | |
| **Information about medication and other treatment options** | Participants’ opinions differed in terms of the comprehensiveness or what areas to cover with regards to medication; however, they strongly agreed that only the most common side effects should be mentioned.  *“It differs. Some patients don’t need much of such information and they are mainly interested in when they will start feeling better, and what the treatment will entail – whether they will feel ill after taking the medication, whether they will be sleepy, gain weight, will be able to drive a car. Some of them are only interested in such practical effects of the treatment, not in biochemistry. But some, and there are fewer of them, are more interested in how the medication works, why some medication can be combined with others, and why it is basically pointless to combine others.” (IP_L)*  *“I would focus on the most important side effects. Like, 1 in 10, 1 in 100.” (IP_A)*  While participants did not want patients modifying their treatment by themselves, participants emphasized including information in the app to inform patients that their doctor could adjust their treatment if it was not working.  *“It is good to give this information. I give this information to the patients at our first meeting. I always tell them that we are going to wait to see if and what effect the treatment will have. I tell them it is possible to change the medication, or it is possible to change psychiatrist, or that they have an opportunity of institutional treatment.” (IP_N)*  *“Very good information. Because sometimes people who are close to suicide say, ‘My doctor cannot help me, then I have nothing. So, there is nothing more.’ They are thinking in this way. ‘My doctor gave me a medication which doesn't help and now I'm ready. Nobody can help me.’ Yeah, for this it would be very useful to say, ‘Look, there are other options.’” (IP_C)* |
| **Information about mental illness** | Participants mentioned that including information for patients to have a better understanding of their diagnosis and potential underlying causes would be helpful.  *“In such app one can give ideas, which idea is behind the symptoms. From the psychological ideas, often maybe people would say, "Ah, this could be true, because of…" and then they go on the level to think about their life. If you are only always on the level of symptoms and body, they will not find a connection to their life.” (IP_C)*  *“Some information about the mental illness.” (IP_M)* |
| **Additional sources of information** | In addition to specific information sections on the app (e.g., medication, illness), interviewees mentioned that it would be helpful to have a section listing and answering common concerns and questions from patients, as well as links to evidence-based, factually accurate websites.  *“Also there could be FAQs. They should read that first and then ask questions that they didn’t find there.” (IP_M)*  *“I think it is good. And perhaps in addition, a link to an evidence-based site that also says it from experts: There is no addiction. Because often people don't believe it.” (IP_A)* |
| **Tracking** | Participants came up with a long list of ideas that may be helpful to monitor (e.g., mood, stress, weight, socialization, etc.; full list of shared ideas is available in Table 1). It was suggested that patients should be able to choose what they want to monitor and that these features could be activated or deactivated as needed.  *“You need appreciation, and you need to know that you are making progress. […]You could see where were you when you downloaded the app, and you could see a month later and you can see two months later, maybe like looking in the retrospective you can see. If you don't note things down, you don't know them.” (IP_F)*  *“I think it would be better if they selected what they want to monitor. It will motivate them better if they select it themselves. But then if they would come for a check-up and I found out they are monitoring things that are not significant, or if I needed them to monitor something else, I would tell them, and we would select it together.” (IP_O)* |
| **Reminders** | A list of common reminders that patients may need was created (e.g., doctor’s appointments) and it was suggested that there be individualization around the types of reminders and number of reminders a patient received.  *“Not only the reminders of medication intake, but also of food and water intake.” (IP_J)*  *“Yeah, I think that would be a very good app. And also to extend the function so that if at the moment I can't take my medicine, I hit the snooze button, or that it will remind me again and again and again.” (IP_A)* |
| **Appointment booking system** | A feature that seemed like it would be helpful for both psychiatrists and patients was an appointment booking system.  *“I’d like to go back to the appointment-making system. I think that if the app was made well, doctors would like to use the app just because of it.” (IP_K)* |
| **Sharing data & reports** | The sharing of information was mentioned in both directions: from the patient to their doctor and family (with regards to app data), and from doctors to the patient (in the form of their medical records).  *“Maybe during the visit, two people, the therapist and the patient, could agree together if we want to share some of the info. And if the patient agrees, then the doctor would have it somewhere in their computer, the access to that info. And if not all the time, I could receive it from you in a period of three weeks, for example. […] I could monitor better and you could monitor better. Because it's a win-win situation for us both.” (IP_F)*  *“Also there could be some pairing with other phones, for example of family members, through the cloud, etc., but the patient must not feel like they are controlled by someone. To whom is the data going to be sent? It could be sent to a doctor.” (IP_K)*  *“We would need some feedback, someone who would reply to the patients, analyse the information, so the patient would have some feedback. First, he would have the impression that someone actually cares about his problems and that he is not using the app pointlessly; it would reflect his mental state.” (IP_H)* |
| **Communication options** | Participants mentioned a variety of communication options that the app could contain, from one-on-one messaging between a patient and their doctors, to forums and chat rooms. In these group communication channels it was suggested that patients could converse with other patients, doctors, and support people (e.g., family, social workers). Connecting patients to a call center for more immediate support was also mentioned.  *“I think a part of the app for medication should be like for questions that could be answered by a professional but also other users.” (IP_E)*  *“Then you have a notification system if someone gets, for instance, suicidal. Then you have a notification system that the therapist will be notified, and you can make a personal chat with him directly and so on.” (IP_D)*  *“There could be some categories: A group for people with depression, another for people with anxiety, etc. They could discuss there. There could be some system of questions and answers: They would ask the questions, a professional would answer, and everyone would see it.” (IP_K)* |
| **Plan out their day** | Participants mentioned that it may be helpful for some patients to create a plan for their day in the app to help keep them on track.  *“I also have many clients who have problems with procrastination. They could make a plan for what to do when, and then follow it and not postpone it for another day.” (IP_J)* |
| **Life path & goals** | A section for big-picture goals was also mentioned as something the app could contain. Participants noted it could be helpful for patients to see themselves achieving goals or even making progress towards the goal.  *“I think this is useful. A depressed patient is hypoactive, so we need him to get back to normal life. Of course, we are adjusting the mood by medication, but we need him to be able to live as before. And it might be good for the patient to see what he has done, what he achieved.” (IP_Q)* |
| **Emergency plan** | Participants recommended the option to create an individualized emergency plan as part of the mobile app, as well as connect it with other app features, such as monitoring. If monitored aspects (e.g., sleep or eating) changed significantly in a negative manner, the patient could receive a ‘warning message’.  *“An emergency plan is a good thing. I think it is customary to make it. Not in detail, but some basic points about what to do in the critical moments. To think in advance and make an emergency plan is nice. Also, if they monitor their functions – sleep, eating, appetite, weight change, etc. The patient might not notice the change in their moods or emotions, but they might notice [the change in the monitored data].” (IP_J)*  *“I think this [emergency plan] would be good. […] In case of emergency, there are some steps, but the device will guide you through the steps. [At] the end […], there will be a call to a professional hotline or something.” (IP_D)*  *“Alcoholics make this at the beginning of their treatment. They make the emergency plan, have allies, phone numbers, who they can contact if something happens […] This could also work for other patients […] But I am not sure whether they would manage to do it by themselves […] maybe in co-operation with some professional who would help them […] Or the app could, through some algorithm, offer some options.” (IP_M)* |
| **Design** | |
| **Simple, easy, not**  **overwhelming** | Participants emphasized a simple and easy handling of the app. It should have a simple user interface and be easy to navigate and understand for both the patient and psychiatrist.  *“It should be plain. It should be simple. Not very complicated.”* *(IP_F)*  *“I think it's really important that it should be an app that is really easy to handle, because all the doctors are not used to it and if there are more than two clicks, they are completely overwhelmed. And if I cannot handle this app, I would not recommend it to a patient. So, first of all, I have to get used to it. And if it's easy and I can check it and if I'm convinced, I will also recommend it to the patient.” (IP_A)* |
| **Positive & motivating** | Participants stressed that the app should be a positive tool in terms of both content and design.  *“The app should not scare the patient. It shouldn’t say that there is a possibility of some risk, there should be no red exclamation marks, etc. It should be a positive tool; it should praise the patient and positively motivate them.” (IP_K)*  *“If somebody can read stories about the same illness from other patients, it could be helpful to read that they had a successful treatment.” (IP_C)* |
| **Pictures** | Pictures were mentioned when discussing the design of the mobile app as a way to make information more digestible and keep patients engaged.  *“Patients like pictures. We could put [pictures] in there of the neuroreceptors, etc., how the stimulation of the neuroreceptor works.” (IP_K)* |
| **Video explanations** | Including videos in the app that cover different topics (e.g., medication) was mentioned in some interviews.  *“You can make videos. But choosing the right video could be done by first talking to a therapist, and then the therapist could say, ‘Okay, I think this video is suited for you. And if afterwards there are still some questions, you could ask me.’” (IP_D)* |
| **Interactive** | Participants mentioned that it was important to have the app be interactive so it would be more engaging for users.  *“Make it a little bit interactive. Let youngsters play a little with it. When I do this, then which branches open up? A little bit of interaction is intriguing. Like, ‘Put your age’, ‘Put your school’, ‘Your study’. […] So, I think if the interactivity is interesting, let's make it a little bit silly. You know, let's play.” (IP_F)* |
| **Story or game** | Along the lines of having an interactive app, the idea of having a story throughout the app or gamifying parts of it arose in the interviews.  *“For children it could be in some form of a game or story with some hero who has some illness as well.” (IP_O)*  *“Make it a little bit like how the video games go to the next level and the next level… Maybe this is goal meeting.” (IP_F)* |
