# Supplementary material for: Recommendations for mobile apps for mental health treatment: Qualitative interviews with psychiatrists
Source: Digit Health. 2025 Mar 17;11:20552076251325951. doi: 10.1177/20552076251325951 (PMC11915247; doi:10.1177/20552076251325951)
Supplement: sj-docx-2-dhj-10.1177_20552076251325951 - Supplemental material for Recommendations for mobile apps for mental health treatment: Qualitative interviews with psychiatrists [file sj-docx-2-dhj-10.1177_20552076251325951.docx]

Fonts:

*Introduction*

**Main question**

-----------------------------------------------------------------------------------------------------------------

Interview Guide

*This study is anonymous. You can disclose anything you have in mind. We appreciate when you share the reality, not what can be found in guidelines.*

*Feel free to take your time to answer the questions and think longer, when you need to.*

*A, Perception of the current system:*

1. **Can you describe the routine procedure in your practice, when it comes to introducing a patient to a new psychiatric medication, in outpatient care?**
2. **What are the gaps in currently practiced methods?**

*B, How to fill the gaps/recommendations and suggestions for improvement:
Our intention for the future is to develop a solution, let’s say a mobile app, which would help psychiatric patients manage their medication. Now, let’s brainstorm about how e-health or new mobile applications could fill some of the above mentioned gaps. Also, I would like you to think about what functionalities (qualities) should a new solution have, in order to be helpful for the patients, to help them to understand their medication and to engage them with their treatment.*

1. **What functionalities do you think a new app should have? / What do you suggest to include in a new app?**
2. **How do you think we can motivate - patients to use the MM app, as something new in their life? - doctors**
3. **Do you have any experience with e-Health, as a user?**
   **if yes, describe it.**
4. **Do you have any additional thoughts or suggestions on how to help patients to manage their psychiatric medication?**
