# Supplementary material for: Recommendations for mobile apps for mental health treatment: Qualitative interviews with psychiatrists
Source: Digit Health. 2025 Mar 17;11:20552076251325951. doi: 10.1177/20552076251325951 (PMC11915247; doi:10.1177/20552076251325951)
Supplement: sj-docx-3-dhj-10.1177_20552076251325951 - Supplemental material for Recommendations for mobile apps for mental health treatment: Qualitative interviews with psychiatrists [file sj-docx-3-dhj-10.1177_20552076251325951.docx]

**Consolidated Criteria for Reporting Qualitative Studies (COREQ): 32-Item Checklist**

| Item | Guide Questions/Descriptions | Page Addressed On/Notes |
| --- | --- | --- |
| Domain 1: Research Team and Reflexivity | | |
| Personal Characteristics | | |
| 1.Interviewer/facilitator | Which author/s conducted the interview or focus group | Page 5 |
| 2.Credentials | What were the researcher’s credentials? | Page 6 |
| 3.Occupation | What was their occupation at the time of the study? | Page 6 |
| 4.Gender | Was the researcher male or female? | Page 6 |
| 5.Experience and training | What experience or training did the researcher have? | Pages 5-6 |
| Relationship with Participants | | |
| 6.Relationship established | Was a relationship established prior to study commencement? | Page 5 |
| 7.Participant knowledge of the interviewer | What did the participants know about the researcher? | Page 6 |
| 8.Interviewer characteristics | What characteristics were reported about the interviewer/facilitator? | Page 6 |
| Domain 2: Study Design | | |
| Theoretical Framework | | |
| 9.Methodological orientation and theory | What methodological orientation was stated to underpin the study? | Page 8 |
| Participant Selection | | |
| 10.Sampling | How were participants selected? | Page 5 |
| 11.Method of approach | How were participants approached? | Page 5 |
| 12.Sample size | How many participants were in the study? | Page 9 |
| 13.Non-participation | How many people refused to participate or dropped out? Reasons? | Page 9 |
| Setting | | |
| 14.Setting of data collection | Where was the data collected? | Page 6-7 |
| 15.Prescence of non-participants | Was anyone else present besides the participants and researchers? | Page 6 |
| 16.Description of sample | What was the important characteristics of the sample? | Page 9 |
| Data Collection | | |
| 17.Interview guide | Were questions, prompts, guides provided by the authors? Was it pilot tested? | Page 6-7 |
| 18.Repeat interviews | Were repeat interviews carried out? If yes, how many? | Page 7 |
| 19.Audio/visual recording | Did the research use audio or visual recording to collect the data? | Page 7 |
| 20.Field notes | Were field notes made during and/or after the interview or focus group? | Page 7 |
| 21.Duration | What was the duration of the interviews or focus group? | Page 7 |
| 22.Data saturation | Was data saturation discussed? | No. Data saturation is not part of Mayring’s structural content analysis methodology. |
| 23.Transcripts returned | Were transcripts returned to participants for comment and/or correction? | No. |
| Domain 3: Analysis and Findings | | |
| Data Analysis | | |
| 24.Number of data coders | How many data coders coded the data? | Page 8 |
| 25.Description of the coding tree | Did the authors provide a description of the coding tree? | No. |
| 26.Derivation of themes | Were themes identified in advance or derived from the data? | Page 8 |
| 27.Software | What software, if applicable, was used to manage the data? | Page 8 |
| 28.Participant checking | Did participants provide feedback on the findings? | Page 9 |
| Reporting | | |
| 29.Quotations presented | Were participant quotations presented to illustrate the themes/findings? Was each quotation identified? | Page 12-16 and in supplementary table S1 |
| 30.Data and findings consistent | Was there consistency between the data presented and the findings? | Page 17-20 |
| 31.Clarity of major themes | Were major themes clearly presented in the findings? | Page 9 |
| 32.Clarity of minor themes | Is there a description of diverse cases or discussion of minor themes? | Page 9-16 and supplementary table S1 |
